# 

**Published:** 1902-08-23

**Authors:** 


### NOTICE TO CORRESPONDENTS.

All MSS., Letters, Books for Review, and other matters intended for the Editor should be addressed The Editor, "The Hospital," The Hospital Building, 28 & 29 Southampton Street, Strand, W.C.

The Editor cannot undertake to return rejected MS., even when accompanied by stamped directed envelope.

**Notice to Advertisers and Subscribers.**

All Advertisements, Orders for Copies of The Hospital, and Business Communications should be addressed to **THE MANAGER, (Not to the Editor)**, The Hospital, 28 & 29 Southampton Street, Strand, London, W.C.

The Cost of Subscription, post free, is as follows.—Per week, 2½d.; per quarter, 2s. 8d.; per half-year, 5s. 3d.; per annum, 10s. 6d. Foreign:—Per annum, 16s. 2d., payable, in all cases, in advance.

**NOTICE.—Vol. XXXI. of "The Hospital" (October 1901, to March 1902), in handsome cloth binding is now on sale at the office of this Journal, price, 7s. 6d. Cases for binding the Numbers contained in this Volume 1s. 6d. Index to Vol. XXXI., 2½d., post free.**

**The Monthly Part for August is now ready. Price 6d., by post 8d.**

### BOOKS RECEIVED.

J. and A. Churchill.

"The Force of Mind or the Mental Factor in Medicine." By Alfred T. Schofield, M.D., M.R.C.S.

Longmans, Green and Co.

"Report of the Yellow Fever Expedition to Pará." By H. E. Durham.

Henry J. Glaisher.

"Small-pox: How it is Spread and How it may be Prevented." By James Wallace, M.A., M.D.Aberd.

W. Hepworth, Kidderminster.

"Prayers for Hospital Wards." Compiled by A. B.

prove of the greatest comfort in special cases; and where money does not matter, and in cases of long and helpless illness, the benefit of a practically designed adjustable frame is incalculable. The "Surcar" bedstead, upon which the makers are to be congratulated, is to be seen at Messrs. J. and A. Carter's showrooms, 6A New Cavendish Street, Portland Place, London, W.

### Scraps and Gleanings.

The sum of £100 has been given by an anonymous donor to the fund for developing the work of the Cancer Research Department of the Middlesex Hospital.

In the town of Warminster, in Wiltshire, the cottage hospital was awarded a prize for the decorations in honour of the King and Queen. The hospital was described by the judges as "a fairy-land" when lighted up.

Mr. Walter Haddon, of Salisbury Square, has promised to subscribe £50 a year for ten years to the fund for creating "Longest Unsuccessful Candidate Pensions," in connection with the Printers' Pension Corporation.

During the past month the work of the inspectors of the National Society for the Prevention of Cruelty to Children included 11,692 visits of supervision, the Society inquired into 3,484 complaints of neglect, etc., of which 3,126 cases were dealt with; 2,988 cases were found to be true.

The Bishop of London appeals, on behalf of the Children's Country Holiday Fund, for £2,000, in order to send to the country or seaside, for a fortnight's holiday, some 12,000 children who have paid the amount required of them, and are now waiting to know it they may go. Unless this £2,000 is forthcoming, all these 12,000 children will have to be disappointed.

The annual report of the Ropner Convalescent Home at Dinsdale shows that during the season of 26 weeks from May to November 364 patients were admitted as compared with 309 in the previous year. The income for the year was £882, being an increase of £26 on the previous year, and the expenditure was £676. The subscriptions from workmen amounted to £680, being an increase of £22 on the last year's subscriptions from this source.

### The Student of Medicine.

### Appointments.

**W. J. M. Ettles**, M.D.Aberd., appointed Medical Officer of Health for No. 11 District of the London County Council. **Charles Fisher**, M.B., B.S.Dur., M.R.C.S., L.R.C.P., appointed Deputy Poor-law Medical Officer and Public Vaccinator to the Albury District of the Guildford Union. **Norman E. Harding**, M.B., Ch.B.Edin., appointed Second House Surgeon to the East Suffolk and Ipswich Hospital. **E. F. G. T. Heap**, M.R.C.S., L.R.C.P.Lond., appointed District Medical Officer of the Grimsby Union. **E. M. Hime**, M.B., Ch.B.Vict., appointed District and Workhouse Medical Officer of the Aysgarth Union. **John Hoole**, M.R.C.S.Eng., L.S.A.Lond., appointed Medical Officer and Public Vaccinator to the Hartington District of the Ashbourne Union. **C. P. Lapage**, M.B., Ch.B., appointed House Surgeon to the Manchester Royal Infirmary. **Gerald Leighton**, M.D., appointed Interim Professor of Pathology and Bacteriology at the Royal Veterinary College, Edinburgh. **W. McWalter**, M.B., appointed Medical Officer to His Majesty's Prison, Peterhead. **T. Hugh E. Meggs**, M.R.C.S., L.R.C.P., appointed Public Vaccinator of the Slough District of the Eton Union. **G. H. Monro-Home**, M.D.Edin., appointed Honorary Anæsthetist to the David Lewis Northern Hospital, Liverpool. **E. Nicholson**, M.R.C.S.Eng., L.R.C.P.Lond., appointed Medical Officer of Health for the Fenny Stratford Urban District Council. **Leslie Paton**, B.A., M.B., B.C.Camb., F.R.C.S.Eng., appointed Assistant Ophthalmic Surgeon to St. Mary's Hospital, Paddington. **W. J. Sweeney**, M.B., B.S., R.U.I., appointed Deputy Medical Officer of the Workhouse and the Thornbury District of the Thornbury Union. **Abraham Thomas**, M.B., B.S.Lond., reappointed Medical Officer of Health to the Aberystwith Urban District Council. **R. C. Verley**, M.B., Ch.B.Edin., M.R.C.S., L.R.C.P., B.Sc., appointed House Physician to the General Hospital, Birmingham. **J. S. Warrack**, M.A., M.D.Aberd., appointed Deputy Medical Officer of Health for Tottenham. **R. Young**, M.B., C.M.Aberd., appointed District and Workhouse Medical Officer of the Rochford Union.

### "The Hospital" Institutional Library.

"Hospitals and Asylums of the World." 4 Vols., with a Portfolio of Plans, complete £12 12s.

"Burdett's Hospitals and Charities, 1902." 5s.

"Cottage Hospitals, General, Fever, and Convalescent." 10s. 6d.

"Hospital Expenditure: The Commissariat." 2s. 6d.

"A Legal Handbook for the Use of Hospital Authorities." 2s. 6d.

"The Cottage Hospital Case Book or Register of Patients." 10s. and 12s. 6d.

"The Furnishing and Appliances of a Cottage Hospital." 6d.

All these are published by the Scientific Press, Ltd., and may be obtained through any bookseller, or direct from the publishers, 28 and 29 Southampton Street, Strand, London, W.C.

